# Detection of inspiratory recruitment of atelectasis by automated lung sound analysis as compared to four-dimensional computed tomography in a porcine lung injury model

**DOI:** 10.1186/s13054-018-1964-6

**Published:** 2018-02-24

**Authors:** Stefan Boehme, Frédéric P. R. Toemboel, Erik K. Hartmann, Alexander H. Bentley, Oliver Weinheimer, Yang Yang, Tobias Achenbach, Michael Hagmann, Eugenijus Kaniusas, James E. Baumgardner, Klaus Markstaller

**Affiliations:** 1 Department of Anesthesia, General Intensive Care Medicine and Pain Management, Medical University Vienna, Waehringer Guertel, 18-20 Vienna, Austria; 2 Department of Anesthesiology, Medical Center of the Johannes-Gutenberg University Mainz, Mainz, Germany; 3 Department of Diagnostic and Interventional Radiology, Medical Center of the Johannes-Gutenberg University Mainz, Mainz, Germany; 4 Center for Medical Statistics, Informatics, and Intelligent Systems, Medical University Vienna, Vienna, Austria; 5 Institute of Electrodynamics, Microwave and Circuit Engineering, Vienna University of Technology, Vienna, Austria; 6 Department of Anesthesiology, University of Pittsburgh Medical Center, Pittsburgh, PA 15261 USA; 7 Department of Diagnostic and Interventional Radiology, University Hospital of Heidelberg, Heidelberg, Germany; 8 Translational Lung Research Center Heidelberg (TLRC), Member of the German Center for Lung Research (DZL), Heidelberg, Germany; 9 Institute of Diagnostic and Interventional Radiology, St. Vinzenz Hospital, Cologne, Germany

**Keywords:** Cyclic recruitment, Lung sounds, Dynamic computed tomography, Atelectasis, Positive end-expiratory pressure

## Abstract

**Background:**

Cyclic recruitment and de-recruitment of atelectasis (c-R/D) is a contributor to ventilator-induced lung injury (VILI). Bedside detection of this dynamic process could improve ventilator management. This study investigated the potential of automated lung sound analysis to detect c-R/D as compared to four-dimensional computed tomography (4DCT).

**Methods:**

In ten piglets (25 ± 2 kg), acoustic measurements from 34 thoracic piezoelectric sensors (Meditron ASA, Norway) were performed, time synchronized to 4DCT scans, at positive end-expiratory pressures of 0, 5, 10, and 15 cmH_2_O during mechanical ventilation, before and after induction of c-R/D by surfactant washout. 4DCT was post-processed for within-breath variation in atelectatic volume (Δ atelectasis) as a measure of c-R/D. Sound waveforms were evaluated for: 1) dynamic crackle energy (dCE): filtered crackle sounds (600–700 Hz); 2) fast Fourier transform area (FFT area): spectral content above 500 Hz in frequency and above −70 dB in amplitude in proportion to the total amount of sound above −70 dB amplitude; and 3) dynamic spectral coherence (dSC): variation in acoustical homogeneity over time. Parameters were analyzed for global, nondependent, central, and dependent lung areas.

**Results:**

In healthy lungs, negligible values of Δ atelectasis, dCE, and FFT area occurred. In lavage lung injury, the novel dCE parameter showed the best correlation to Δ atelectasis in dependent lung areas (R^2^ = 0.88) where c-R/D took place. dCE was superior to FFT area analysis for each lung region examined. The analysis of dSC could predict the lung regions where c-R/D originated.

**Conclusions:**

c-R/D is associated with the occurrence of fine crackle sounds as demonstrated by dCE analysis. Standardized computer-assisted analysis of dCE and dSC seems to be a promising method for depicting c-R/D.

**Electronic supplementary material:**

The online version of this article (10.1186/s13054-018-1964-6) contains supplementary material, which is available to authorized users.

## Background

Although positive pressure ventilation can be life-saving by restoring adequate oxygenation, mechanical ventilation itself can lead to secondary lung damage [1, 2]. In addition to volutrauma and barotrauma, atelectrauma (cyclic recruitment and de-recruitment of atelectasis, or c-R/D) also contributes to ventilator-induced lung injury (VILI) [2, 3].

Numerous studies have investigated c-R/D and addressed the specific role of atelectrauma in experimental settings. Using dynamic computed tomography (dCT), within-breath recruitment and de-recruitment were visualized by variations in atelectatic lung fractions [4, 5]. Further experimental studies demonstrated that c-R/D leads to respiration-dependent oscillations in blood oxygenation that originate in the lungs [6] and are forwarded downstream via the circulation to the end-organ level [7, 8]. In this context, more severe lung tissue damage and an increased inflammatory response have been shown in lung areas where c-R/D occurs [9, 10], highlighting the relevance of c-R/D to the onset of VILI.

Recently, several novel ventilatory strategies have been proposed for the purpose of avoiding c-R/D during mechanical ventilation [11–13], In clinical practice, however, bedside detection of the dynamic process of c-R/D is not possible with currently available tools.

A noninvasive, bedside method that might be adapted for the detection of c-R/D is automated lung sound auscultation [14, 15]. The first attempt to assess tidal recruitment by automated lung sound analysis was presented by Vena and colleagues. They post-processed an acoustic parameter that reflects the changes in spectral characteristics of lung sounds during inspiration [16], termed “fast Fourier transform area” (FFT area).

Our study focused on the technical development of a novel sound-based parameter for the detection of within-breath recruitment, in a model where the within-breath changes in atelectasis (Δ atelectasis) could be verified by the reference method of four-dimensional computed tomography (4DCT). For our investigations, we proposed to induce a broad range of c-R/D conditions by setting different positive end-expiratory pressure (PEEP) levels at a fixed end-inspiratory pressure level of 30 cmH_2_O, resulting in different tidal volumes. In this setup, we aimed to capture and quantify the distinct sound signature (i.e., adventitious sounds) associated with the sudden opening of atelectatic lung units during inspiration by post-processing the “dynamic crackle energy” (dCE) in the frequency range of 600 to 700 Hz. Moreover, we aimed to localize the origin of c-R/D acoustically by assessing the “dynamic changes in spectral coherence” (dSC) throughout inspiration.

As such, we hypothesized that there is a linear correlation between Δ atelectasis and dCE, and Δ atelectasis and the reproduced FFT area parameter, respectively. Additionally, we hypothesized that dSC is different in regard to different lung regions and PEEP levels.

## Methods

### Animal experiments

Following Animal Care Committee approval (Landesuntersuchungsamt Koblenz) of the Rhineland Palatinate, Germany (23,177-07/G09-1-029), 10 piglets were studied. One animal was needed to set up the protocol. Two animals expired during c-R/D induction and one did not provide a complete dataset due to technical failures. Thus, six animals were included in the final analysis. All procedures were performed under deep anesthesia, and careful efforts were made to minimize suffering.

After induction of general anesthesia, catheters (for the purposes of invasive monitoring) were surgically placed. Details concerning the anesthetic procedures and routine monitoring regimen can be found in Additional file 1.

### Preliminary experimental tests

Before carrying out the study, we assessed the influence that surrounding noise might exert upon the attached piezoelectric contact sensors, and the influence the sensors themselves might exert upon the radiologic imaging quality. Using a noise-absorbing mat that we wrapped around the subjects, pretesting showed that the recorded raw data sound waveforms were not noticeably affected by external noise. Furthermore, no specific artifacts could be attributed to the sensor positioning. Concerning the metallic acoustic sensors themselves, we found that they produced a bias of up to 40 Hounsfield Units (HU) on mean lung densities (MLDs) in computed tomography (CT) imaging (see Additional file 1: Figure S1).

### Automated lung sound recordings and four-dimensional computed tomography

**Fig. 1:**
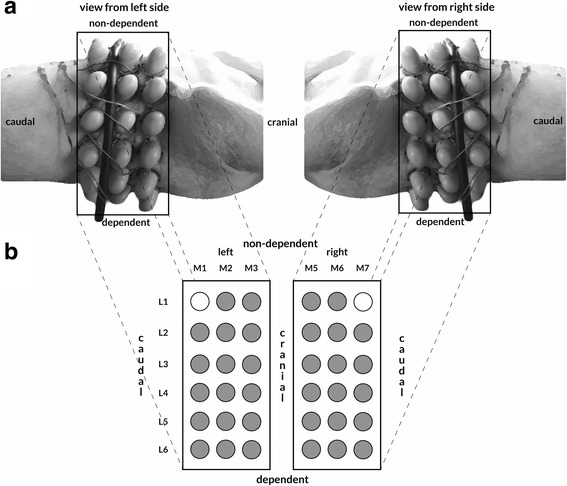
Anatomical sensor positions. **a** Lateral views of the thorax of one exemplary investigational subject, demonstrating the anatomical positions of the acoustic sensors attached. **b** The respective sensor matrix used

In our experimental setup, 36 piezoelectric contact sensors (Meditron ASA, Oslo, Norway) were arranged into two arrays (one on the left side and one on the right), with each array containing 18 sensors organized into three columns (M1–M3) and six rows (L1–L6). Two of the 36 sensors were inactive and served as a reference for ambient noise. Figure 1 demonstrates the anatomical sensor positions. The sensor arrays were placed in a circular fashion around the pig’s thorax using special gel pads, thus forming apical (columns M3 and M5), middle (columns M2 and M6), and basal (columns M1 and M7) transversal sensor planes. Correspondingly, the first and second rows of sensors (L1 and L2) covered the nondependent lung areas, the third and fourth rows (L3 and L4) covered the central lung areas, and the fifth and six rows (L5 and L6) covered the dependent lung areas. Placement of the sensor arrays was CT-guided so as to position the basal transversal sensor plane 2.5 cm above the dome of the diaphragm. Based on the predefined sensor matrix, the sensors of the middle transversal sensor plane were located between the orifice of the upper right and middle right lung lobes, while the apical transversal sensor plane was located roughly at the bifurcation of the trachea. The sensor arrays were connected to the vibration response imaging (VRI) device (VRIxv, GE Healthcare, Little Chalfont, UK) and raw data acoustic waveforms were collected at a sampling frequency of 19,200 Hz.

4DCT measurements (Brilliance iCT 256-slice scanner, Philips, Amsterdam, the Netherlands) were performed on identical lung regions with a cranio-caudal span of 8 cm, correlating directly with the placement of the sensor array matrix. In accordance with the anatomical positions of the acoustic sensors, nondependent, central, and dependent lung regions were analyzed.

### Study protocol

Measurements of lung sound acoustics were performed which were time-synchronized to 4DCT at randomly set PEEP levels of 0, 5, 10, and 15 cmH_2_O during both healthy baseline (BLH) and after induction of the model lung injury (surfactant depletion injury (LAV)).

To study c-R/D, surfactant depletion was induced via repetitive lung lavages using isotonic solution (30 ml/kg) until reaching a lung state characterized by substantial lung collapse (defined as a Horowitz-index < 300 at zero end-expiratory pressure (ZEEP)), but still retaining the capability of partial within-breath recruitment (defined as Horowitz-index < 450 at a PEEP of 15 cmH_2_O). This model was similar to that used in one of our previously published studies [13]. A pressure controlled ventilation (PCV) regimen was chosen. To produce a broad range of c-R/D conditions, we used different PEEP levels (so as to vary static recruitment) at a fixed end-inspiratory pressure of 30 cmH_2_O, and an inspiration-to-expiration ratio of 1:1. This resulted in different driving pressures and different tidal volumes for the investigation of within-breath recruitment, while keeping the mechanism of recruitment unchanged.

Each PEEP level was maintained for at least 10 min; then, data were recorded for a period of 20 s at a respiratory rate of 6 breaths/min due to the limited temporal resolution of the CT scanner.

### Offline data handling of four-dimensional computed tomography scans

Similar to the methodology utilized in a previous study [17], quantitative analysis of CT attenuation of lung tissue was carried out semi-automatically using an in-house-developed software (YACTA version 1.09.40, University of Mainz, Germany), which was written by one of the authors (OW). Details about 4DCT post-processing are provided in the supplemental section (Additional file 1: Figure S2). The behavior of atelectatic (−300 to 0 HU), poorly aerated (−600 to −301 HU), normally aerated (−900 to −601 HU), and hyperinflated (−1024 to −901 HU) lung volumes were computed over the time course of the breathing cycle in steps of 0.58 s. The amount of c-R/D was evaluated by assessing the differences between end-expiratory and end-inspiratory values in the atelectatic lung volume (Δ atelectasis). An example of 4DCT post-processing appears in the supplemental section (Additional file 1: Figure S3).

### Offline data handling of automated lung sound recordings: overview

Raw data sound waveforms were post-processed to evaluate three different parameters.

The first parameter was dynamic crackle energy (dCE). This parameter reflects the amount of sound energy in the frequency spectrum of 600 to 700 Hz over the inspiratory time course of the breathing cycle [14, 18, 19]. According to the literature, this is the defined frequency band of fine crackle sounds [20–22].

For the second parameter, we reproduced the fast Fourier transform (FFT) analysis introduced by Vena et al. [16] (termed FFT area) by calculating the spectral content above 500 Hz in frequency and above −70 dB in amplitude in proportion to the total amount of sound above −70 dB amplitude.

The third parameter was spectral coherence (SC)/dynamic spectral coherence (dSC). These parameters reflect regional acoustical homogeneity in the subjacent lung regions of neighboring acoustic sensors (SC) and their variation over the inspiratory time course (dSC) [23].

Essentially, the first two parameters were used to search for a linear correlation between Δ atelectasis and dCE, and Δ atelectasis and FFT area, while the purpose of the dSC parameter was to localize the origin of within-breath recruitment acoustically.

**Fig. 2:**
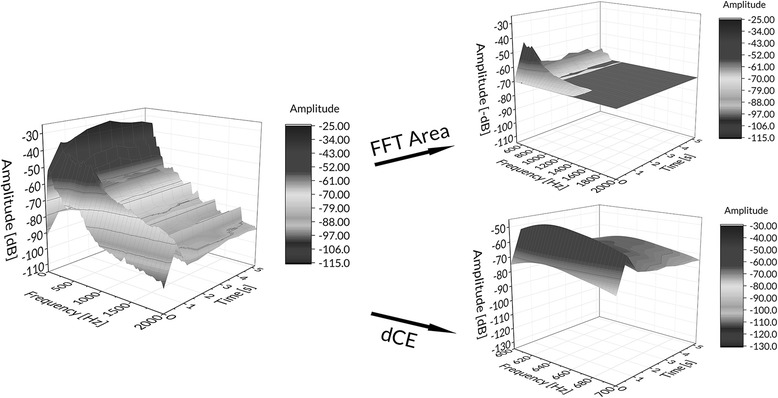
Post-processing of recorded acoustic lung sounds. An example of automated lung sound analysis for “dynamic crackle energy” (dCE) and “fast Fourier transform area” (FFT area). The three-dimensional plots show the amplitude-frequency spectrum over time of one inspiratory cycle. The left side displays the raw data recordings; the right side displays the results after filtering. For calculation of FFT area parameter, the sound spectral content above 500 Hz in frequency and above −70 dB in amplitude in proportion to the total amount of sound above −70 dB amplitude was assessed (upper right); for the dCE parameter, the sound energy in the frequency spectrum of 600 to 700 Hz was post-processed (lower right)

An example of the data handling of acoustic lung sounds is given in Fig. 2.

### Details of the automated lung sound analysis

All analyses were primarily carried out for each acoustic sensor and in time clips of 0.58 s to match the demand acquisition time of 4DCT scanning, resulting in paired measurements over the entire breathing cycle. For final statistics, results were subsumed for the inspiratory phase of the entire (global) lung, and regionally for dependent, central, and nondependent lung areas, by summarizing the parameter values of the respective acoustic sensors overlaying the defined lung regions (see Fig. 1). All computing was performed using the programming environment MATLAB and Simulink Toolbox Release 2014b (The MathWorks, Inc., Natick, MA, USA).

### Assessment of the dynamic crackle energy (dCE)

The dCE parameter was computed as follows. Raw data sound waveforms were downsampled by a factor of 4 from 19,200 Hz to 4800 Hz. Then, fine crackle sounds were isolated using a band-pass filter with finite impulse response (FIR; two-hundredth order with the cut-off frequencies of 600 and 700 Hz). In a subsequent step, for each audio clip of 0.58 s, the root mean squares (RMS) of downsampled sound amplitudes were calculated.

### Assessment of the spectral characteristics of lung sounds (FFT area)

For the assessment of the FFT area, the following operations were performed. After downsampling to 4800 Hz, the signals were bandpass filtered (FIR-filter of two hundredth order in the frequency range of 75–2000 Hz). For each audio clip of 0.58 s, the power spectral densities of the signals were computed (using Welch’s method to convert the signals from the time to the frequency domain by Fourier transform: 200 samples window length, 50% overlap, Hamming-windowing) and transformed to decibels (dB). Using the resulting graph (Additional file 1: Figure S4), the area under the curve (AUC) above −70 dB in amplitude and above 500 Hz in frequency was related back to the AUC above −70 dB and expressed as a percentage.

### Assessment of regional spectral coherence (SC) and its variation over time (dSC)

To localize the origin of c-R/D acoustically, the spectral coherence method was used (representing a function of frequency that indicates how well two sounds match at each frequency, i.e., the better the match, the better the homogeneity of sounds from adjacent lung regions). Raw data waveforms were downsampled (4800 Hz) and bandpass filtered (75–2000 Hz). Then, the signals of neighboring acoustic sensors were analyzed in regards to their mutual spectral coherence. For each lung region of interest (ROI), the arithmetic mean of all pairs of acoustic sensors overlaying the predefined lung regions was assessed in time clips of 0.58 s. From the resulting spectral coherence time plot, two parameters were computed: the spectral coherence (SC) by averaging all values over the inspiratory phase, and the dynamic spectral coherence (dSC) by assessing the time-dependent variation over the inspiratory phase of the breathing cycle (Additional file 1: Figure S5). A more detailed description of how SC and dSC were computed is available in Additional file 1.

### Statistics

The relationships between Δ atelectasis by 4DCT and dCE and FFT area, respectively, were analyzed using linear mixed models (LMMs). These were fitted for the entire region of interest (global), and regionally for nondependent, central, and dependent lung areas (dCT as nondependent variable; dCE and FFT area as dependent variables; piglet ID as random intercept to account for the structure of dependency due to repeated measures; Bonferroni-Holm method for multiple testing). Based on the model intercepts and slopes, the corresponding regression lines and the marginal *R*^2^ were computed [24]. Differences in dSC in regard to lung region (nondependent, central, dependent) and in regard to PEEP (0 and 15 cmH_2_O), were addressed by another LMM, which also tested the interaction between lung region and PEEP. We accounted for the structure of dependency due to repeated measures (Piglet ID as random intercept) and adjusted for multiple testing by the Bonferroni-Holm method.

For descriptive statistics, mean and standard deviation values are reported. Statistics were performed using the statistical software R (R: A Language and Environment for Statistical Computing, R Core Team, R Foundation for Statistical Computing, Vienna, Austria), GraphPad Prism v6 (GraphPad Software Inc., San Diego, CA, USA), and Origin (OriginLab, Northampton, MA, USA).

## Results

### Results of healthy baseline measurements

Under healthy conditions, c-R/D was not evident on 4DCT in any of the animals. BLH measurements found a negligible amount of atelectasis (ranging from 1.6 to 18.2 cm^3^) with Δ atelectasis ranging from 0 to 13.3 cm^3^ (ZEEP to PEEP, 15 cmH_2_O). The total volume of the lung stack examined by 4DCT was 295 ± 24 cm^3^, yielding a volume percentage change in atelectasis ranging from 0 to 4.5% of the lung volume imaged. In the synchronous recorded sound waveforms, post-processed values for dCE were minimal, ranging from 0.019 to 0.041, and for FFT area from 3.6 to 13.8% for all PEEP steps and lung regions. Overall, under baseline conditions, the analysis of SC showed high values (56 ± 4.7), with minimal changes over the time-course of inspiration, resulting in dSC values of 2.2 ± 0.5.

### Results of model lung injury measurements

Induction of lung injury by 3 ± 1 lavages induced c-R/D in all subjects, as confirmed by 4DCT. Ventilatory, gas exchange, and hemodynamic parameters are presented in Table 1. Routinely, the highest Δ atelectasis occurred at ZEEP. Within-breath changes in atelectasis decreased with increasing PEEP at the predefined ventilator settings. The variation of PEEP levels (0, 5, 10, and 15 cmH_2_O) at a fixed end-inspiratory pressure of 30 cmH_2_O induced a large range of Δ atelectasis values (from 5.8 to 60 cm^3^). This represented a lung volume change in atelectasis ranging from 1.8 to 20.3% of the imaged lung volume. Table 2 summarizes the atelectatic lung volumes and their changes over the respiratory cycle for the defined lung regions. The full set of post-processed lung volumes are presented in Additional file 1: Figure S6, Tables S1 and S2.

**Table 1:** Ventilatory, gas exchange, and hemodynamic parameters

	LAV 0	LAV 5	LAV 10	LAV 15
P_endinsp_ (cmH_2_O)	29 ± 3	30 ± 3	30 ± 3	30 ± 3
PEEP (cmH_2_O)	0 ± 0	5 ± 1	10 ± 1	15 ± 1
RR (min^−1^)	6	6	6	6
V_T_ (ml)	529 ± 68	517 ± 83	451 ± 64	402 ± 72
Crs (ml/cmH_2_O)	20 ± 4	21 ± 3	23 ± 4	23 ± 4
Flow (L/min)	51 ± 4	52 ± 3	50 ± 5	50 ± 5
F_I_O_2_	1.0	1.0	1.0	1.0
P_a_O_2_ (mmHg)	248 ± 131	287 ± 107	381 ± 147	412 ± 153
P_a_CO_2_ (mmHg)	46 ± 12	45 ± 10	47 ± 11	48 ± 12
SpO_2_ (%)	98 ± 2	99 ± 1	99 ± 1	99 ± 1
HR (min^−1^)	97 ± 28	95 ± 26	108 ± 31	114 ± 35
MAP (mmHg)	78 ± 14	81 ± 19	72 ± 15	68 ± 11
MPAP (mmHg)	37 ± 6	34 ± 7	35 ± 8	33 ± 5
CVP (mmHg)	15 ± 3	16 ± 3	17 ± 5	19 ± 4

Values are given as mean ± standard deviation (SD) for the defined time points in the lavage-injured lungs for the respective PEEP settings of 0 (LAV 0), 5 (LAV 5), 10 (LAV 10), and 15 (LAV 15) cmH_2_O

*Crs* compliance of the respiratory system, *CVP* central venous pressure, *F*_*I*_*O*_*2*_ inspiratory fraction of oxygen, *Flow* airway flow, *HR* heart rate, *MAP* mean arterial pressure, *MPAP* mean pulmonary arterial pressure, *P*_*a*_*CO*_*2*_ arterial partial pressure of carbon dioxide, *P*_*a*_*O*_*2*_ arterial partial pressure of oxygen, *PEEP* positive end-expiratory pressure, *P*_*endinsp*_ end-inspiratory pressure, *RR* respiratory rate, *SpO*_*2*_ peripheral saturation, *V*_*T*_ tidal volume

**Table 2:** Amount of atelectatic lung volumes as measured by four-dimensional computed tomography

	Mean ± SD volume (cm^3^)	
	Atelectasis	Δ Atelectasis
Entire lung stack		
LAV 0	72.95 ± 14.11	37.19 ± 11.94
LAV 5	49.3 ± 14.24	32.6 ± 10.46
LAV 10	39.78 ± 14.2	19.75 ± 5.34
LAV 15	36.84 ± 14.83	11.81 ± 5.04
ROI: non-dependent lung		
LAV 0	9.82 ± 4.73	1.61 ± 1.19
LAV 5	8.74 ± 6.08	1.69 ± 0.42
LAV 10	7.96 ± 5.11	1.26 ± 0.64
LAV 15	8.11 ± 5.4	1.17 ± 0.38
ROI: central lung		
LAV 0	24.81 ± 11.7	7.63 ± 2.56
LAV 5	16.92 ± 9.27	6.98 ± 1.93
LAV 10	15.43 ± 8.88	3.81 ± 2.05
LAV 15	15.63 ± 8.95	3.31 ± 2.17
ROI: dependent lung		
LAV 0	38.32 ± 4.44	28.27 ± 11.47
LAV 5	23.64 ± 4.08	23.93 ± 8.59
LAV 10	16.4 ± 5.83	14.7 ± 4.21
LAV 15	12.59 ± 3.72	7.34 ± 2.93

Results are displayed for the average volume of atelectatic lung for the entire breath cycle (atelectasis) and for the within breath changes in atelectatic volumes (Δ atelectasis) during on-going mechanical ventilation in the lavage-injured lungs (LAV)

Measures are given for the entire lung stack and for nondependent central and dependent lung regions of interest (ROI) itemized for the respective PEEP settings of 0, 5, 10, and 15 cm H_2_O (LAV 0–15)

**Fig. 3:**
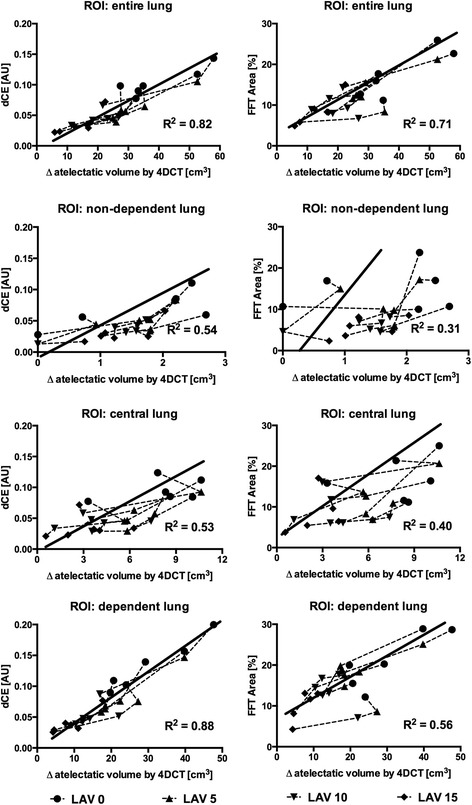
Statistical results of the linear mixed models (LMMs) of the acoustical parameters of “dynamic crackle energy” (dCE; sound energy in the frequency spectrum of 600 to 700 Hz) and “fast Fourier transform area” (FFT area; sound spectral content above 500 Hz in frequency and above −70 dB in amplitude in proportion to the total amount of sound above −70 dB amplitude) versus the within-breath change in atelectatic lung volume (Δ atelectasis) as assessed by four-dimensional computed tomography (4DCT). Plots are presented for different lung regions of interest (ROI) and represent the measurements of all subjects (*n* = 6) in the lavage-injured lungs (LAV). For each investigational subject, the dependent measures are highlighted with respect to the positive end-expiratory pressure (PEEP) levels of 0, 5, 10, and 15 cmH_2_O (LAV 0–15; dashed lines). The solid lines represent the estimated regression lines; *R*^2^ is the computed marginal *R*^2^

Automated lung sound analysis found that adventitious sounds in the frequency range between 600 and 700 Hz (dCE) occurred synchronous to a shift in sound spectral characteristics above −70 dB in amplitude and above 500 Hz in frequency (FFT area) in the presence of c-R/D. Both dCE and FFT area exhibited the highest values at ZEEP, which were reduced when Δ atelectasis decreased as PEEP was increased. For the entire lung region, and for each subregion examined, the dCE and the FFT area varied with Δ atelectasis, with higher correlations for each lung region for the dCE analysis. The detailed results of the LMM analyses with regard to the level of PEEP, as well as the analyzed region, are shown in Fig. 3.

**Fig. 4:**
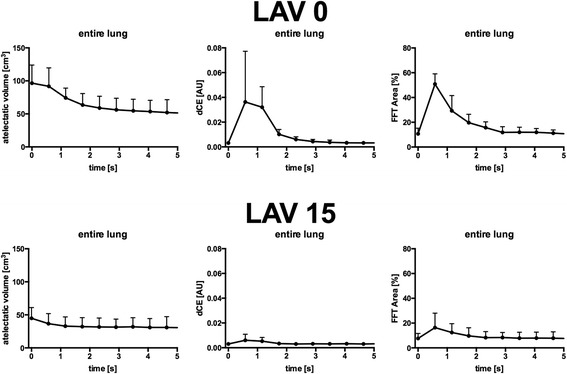
Inspiratory changes in atelectatic lung volume and in the time-synchronized acoustical measures. Plots represent the time-dependent changes over the inspiratory cycle for the atelectatic lung volumes as assessed by four-dimensional computed tomography (4DCT; left column), the acoustical “dynamic crackle energy” (dCE) parameter (middle column), and the reproduced “fast Fourier transform area” (FFT area) parameter by Vena et al. (right column). Data are presented descriptively as mean ± standard deviation (SD) for all subjects (*n* = 6) in the lavage-injured lungs (LAV). The upper row shows the results at a positive end-expiratory pressure (PEEP) of zero (LAV 0); the lower row shows the results at a PEEP of 15 cmH_2_O (LAV 15) for the entire lung stack

Interestingly, dCE and FFT area signals predominately arose in the first 1 to 2 s after the initiation of inspiration, a time period when 4DCT indicated the greatest changes in atelectatic lung volume (Fig. 4). The full dataset is presented in Additional file 1: Figure S7.

The LMM analysis of spectral coherence found that PEEP (*P* = 0.0031) and lung ROI (*P* = 0.0002) had a significant influence on dSC, whereas their interaction (PEEP × ROI) was not significant (*P* = 0.2633). Estimates with standard error of the pair-wise comparisons of the fitted analysis of variance model are presented in Additional file 1: Table S3. We found that dSC significantly differed between dependent and nondependent lung regions (*P* < 0.0001), as well as between dependent and central lung regions (*P* = 0.0067). No significant effect was found between central and nondependent lung regions (*P* = 0.0696). As investigated by variation of PEEP, dSC values decreased from 3.8 ± 0.6/5.7 ± 2.4/8.1 ± 2.4 (nondependent/central/dependent, respectively) at ZEEP to 3.2 ± 0.4/3.9 ± 1.4/5.3 ± 2.4 at a PEEP of 15 cmH_2_O, while SC values increased from 33.3 ± 1.4/36.5 ± 4.2/37.6 ± 4.2 at ZEEP to 42.9 ± 4.1/49.3 ± 4.4/54.5 ± 9.6 at a PEEP of 15 cmH_2_O (Additional file 1: Figure S8).

## Discussion

The present study assessed the potential of automated lung sound analysis for quantifying inspiratory recruitment of atelectasis during mechanical ventilation. Computer-assisted analysis of lung sound recordings could potentially provide continuous, noninvasive, bedside detection of c-R/D with no known hazards (e.g., exposure to ionizing radiation). We used an experimental model of lung lavage to produce surfactant depletion and facilitate c-R/D [13], and we used differing levels of PEEP to vary the amount of c-R/D over a broad range. 4DCT, covering a thick axial lung segment, was used as a standard to define the amount of tidal recruitment of atelectasis. We used one previously reported method [16], as well as two new methods, for analyzing lung sounds to identify and localize within-breath recruitment of atelectasis. Our study showed promising correlations between these sound analysis methods and the amount of tidal recruitment, as assessed by within-breath variation in atelectatic volume by the reference method of 4DCT.

Since the introduction of the stethoscope by Laennec [25], clinicians have used qualitative sound analysis as a diagnostic tool to identify lung pathologies. More recently, automated recording systems have become available that can capture, store, and analyze lung sounds quantitatively to provide further information [26, 27]. Automated lung sound analysis is noninvasive, observer-independent, and allows for an objective measurement and classification of acoustic pattern at standardized conditions. Despite this, its application in a clinical setting is limited due to multiple sources of ambient noise that might bias the results, even though electronic auscultation has the advantage of signal amplification and ambient noise reduction. Although we used a commercially available multisensing technology to record acoustic waveforms, our evaluations were performed on a raw data level, focusing on the detection of adventitious sounds associated with c-R/D. We used the VRI system simply as a methodological tool to standardize lung recordings via a proven, state-of-the-art piezoelectric multisensor recording technology. The idea that c-R/D may generate a distinct sound signature was based on prior studies that attributed fine crackles—defined as short, nonmusical, explosive sounds, typically hovering around a frequency of 650 Hz [18]—to airway opening [19]. Thus, we aimed to capture and analyze sound waves in an experimental model where cyclical recruitment of atelectasis was documented by 4DCT as the reference method.

Our data showed that c-R/D was associated with the occurrence of adventitious crackle sounds during mechanical ventilation. The novel dCE parameter presented here could quantify these crackles and correlated well to within-breath variation in atelectatic volume as assessed by 4DCT. The best dCE results (*R*^2^ = 0.88) were found by the acoustic sensors overlaying the dependent lung regions where c-R/D originated. Additionally, the FFT area analysis (reproduced from Vena et al. [16]) was also well suited to detecting c-R/D when analyzing all acoustic sensors (*R*^2^ = 0.72), but was less useful for regional discrimination (*R*^2^ = 0.31–0.56). Overall, less tight correlations by FFT area (R^2^ = 0.31–0.72) were found when compared to dCE (R^2^ = 0.56–0.88).

To identify the regions of the lung where c-R/D was taking place, we computed the time-dependent variation of spectral coherence of neighboring acoustic sensors during inspiration. Our data showed that highest dSC values occurred in the dependent lung regions and at ZEEP, which was in agreement with 4DCT results, showing that the phenomenon of c-R/D took place at the functional border of atelectatic and poorly ventilated lung compartments. Additionally, increasing the PEEP led to a significant reduction in dSC, while absolute SC values increased, which might be best explained by the restoration of lung homogeneity.

Our study also suggests that acoustic methods can provide some insights into the kinetics of recruitment. The variation in time of dCE and FFT area analysis suggests that the majority of intratidal recruitment took place in the first 1 to 2 s, in agreement with the time course of changes in atelectasis as assessed by 4DCT. Similar time constants during inflation have been reported previously [5, 13, 28].

One of our study’s strengths was the use of 4DCT with a longitudinal coverage of 8 cm as the reference standard. Additionally, the multisensor sound recording system allowed for regional subanalyses. The 4DCT and acoustic methods were precisely time-aligned for all measurements. The study design included large tidal volumes and randomly varying PEEP levels that allowed for observations over a wide range of c-R/D. Our ventilation regimen, however, was not intended to mimic a clinical scenario, but to experimentally induce a large range of c-R/D. Additionally, the acquisition time for 4DCT restricted our study to very slow respiratory rates, although from a technical point of view the dCE parameter could be processed at any physiological respiratory rate. Considering this, we used sharp borders to quantify the amount of cyclic atelectasis, and since the metallic sensors per se had an influence on CT attenuation, the chosen HU range might in part include poorly aerated lung tissue. Moreover, we cannot exclude the possibility that the noise present in the CT signal (which may have been due to these sensors) and the interference due to movement during mechanical ventilation might have biased the weights of the results and statistics. Our study is an evaluation of the potential that sound analysis has to monitor cyclical recruitment of atelectasis, and as such it was carried out in a carefully defined laboratory setting with minimal surrounding noise. Many issues would need to be addressed in order to translate this potential into bedside clinical practice. Although we do not as of yet claim a direct clinical application for our results, this does not preclude consideration of the basic mechanistic concepts for eventual clinical use, possibly even a solution which combines multiparametric lung sound analysis with other noninvasive bedside technologies for the assessment of lung function (e.g., electrical impedance tomography). Concurrent pathophysiologic processes could generate competing lung sounds or alter sound transmission in ways that obscure the distinct sound signatures of inspiratory recruitment, e.g., bronchospasm, fibrotic lung changes, and pneumonia [29–33]. Moreover, the present work cannot define to what extent changes in tidal volume bias the detection of the presented dCE parameter. Thus, one could claim that the reported decrease in Δ atelectasis could be the effect of a decrease in tidal volume rather than a decrease in cyclic lung units opening and closing. We cannot completely exclude this possibility due to our study design (which used varying tidal volumes instead of constant tidal volumes). We believe that this is unlikely, as the FFT area analysis is not dependent upon tidal volume, and both FFT area and dCE yielded high linear correlations for the entire lung stack throughout the various PEEP levels and tidal volumes. Finally, as can be appreciated from Fig. 1, current technology for multisensory sound detection is a bit cumbersome for use in the clinical setting in mechanically ventilated patients, although the technology has been successfully applied in other clinical settings. Although the initial parameter calculations were performed offline—which admittedly was quite time-consuming—the necessary post-processing steps were then converted into a fully automated MATLAB routine that is suitable for integration into any automated lung sound device. Using this script running on a computer with MATLAB, the lung sound parameter calculations take mere seconds, do not require any operator intervention, and provide for the possibility of real-time analysis. The next step would certainly be the implementation of those algorithms via software updates into current automated lung sound devices. Concerning the practicality of lung sound analysis in routine clinical use, we acknowledge that the relevant technology is in need of further development in its current form. Current systems, some of them having been in development for decades now, are not yet streamlined or “simplified” enough for the clinical bedside setting. Nonetheless, these technologies must be continually revisited and enhanced with the latest technological developments (i.e., noise canceling, sensor miniaturization, systems integration, etc.) so as to eventually produce devices and procedures that may be of everyday clinical use, beyond what is currently considered to be possible.

## Conclusions

c-R/D is associated with the occurrence of fine crackle sounds as demonstrated by dCE analysis. Standardized computer-assisted analysis of dCE, in combination with dSC analysis, seems to be a promising method for depicting c-R/D, as shown in an experimental model of surfactant-depleted pigs. Overall, this method was found to be superior to FFT area analysis. The novel parameters presented here for the purpose of acoustical quantification of c-R/D, however, warrant and require further clinical study under realistic conditions before these experimental findings might be translated into clinical practice.

## Additional file

Additional file 1: Supplemental material and supporting information (containing the supplemental Figure S1–S9 and the supplemental Tables S1–S3). (PDF 8554 kb)

## Abbreviations

4DCT: Four-dimensional computed tomography
AUC: Area under the curve
BLH: Healthy baseline conditions
c-R/D: Cyclic recruitment and de-recruitment of atelectasis
CT: Computed tomography
dCE: Dynamic crackle energy
dCT: Dynamic computed tomography
dSC: Dynamic spectral coherence
FFT: Fast Fourier transform
FIR: Finite impulse response
HU: Hounsfield Units
LAV: Surfactant depletion injury
LMM: Linear mixed model
PEEP: Positive end-expiratory pressure
ROI: Region of interest
SC: Spectral coherence
VILI: Ventilator-induced lung injury
VRI: Vibration response imaging
ZEEP: Zero end-expiratory pressure
