# Supplementary material for: Detection of inspiratory recruitment of atelectasis by automated lung sound analysis as compared to four-dimensional computed tomography in a porcine lung injury model
Source: Crit Care. 2018 Feb 24;22:50. doi: 10.1186/s13054-018-1964-6 (PMC6389194; doi:10.1186/s13054-018-1964-6)
Supplement: Supplementary file 1 — Supplemental material and supporting information (containing the supplemental Figure S1–S9 and the supplemental Tables S1–S3). (PDF 8554 kb) [file 13054_2018_1964_MOESM1_ESM.pdf]

Additional file 1 – Supplemental Material

Supplemental Materials and Methods

Details of Anaesthesia and Monitoring

Anaesthesia and monitoring procedures were performed similar to a previous report from our research group [1]. Prior to the experiment, the animals were sedated via intramuscular ketamine (8 mg/kg) and midazolam (0.2 mg/kg). General anaesthesia was induced intravenously with a bolus of fentanyl (4 µg/kg), propofol (4 mg/kg) and pancuronium (0.15 mg/kg) via the ear vein, and the animals were orotracheally intubated (inner diameter of 8.0 mm). Mechanical ventilation was performed using pressure-controlled mode (PCV) (Servo 900C, Siemens, Erlangen, Germany) for all surgical procedures and in between measurements: end-inspiratory pressure ($P_{endinsp}$) was adjusted to achieve a tidal volume (V_T) of 6 ml/kg; positive end-expiratory pressure (PEEP) was set to 5 cm H_2O; respiratory rate (RR) was adjusted to produce an arterial partial pressure of carbon dioxide ($Paco_2$) of 35-40 mmHg; inspired oxygen fraction (F_{IO_2}) was set to 0.3. Anaesthesia was maintained via continuous infusions of propofol (8-12 mg/kg/h) and fentanyl (0.1-0.2 mg/h) for the entire experiment. To maintain fluid homeostasis, 2-4 mL/kg/h of crystalloid infusion (Sterofundin, B.Braun, Melsungen, Germany) was continuously administered. Vascular access for catheter placement was achieved by surgical cut down. An arterial line was placed via the left or right femoral artery. A central venous line via femoral vein and an introducer via right jugular vein for pulmonary artery catheter placement were positioned. Spirometric and hemodynamic parameters (RR, V_T, $P_{endinsp}$, mean airway pressure (M_{PAW}), PEEP, I:E, airway flow, heart rate (HR), mean arterial pressure (MAP), mean pulmonary artery pressure (MPAP) and respiratory system compliance (C_{dyn}) were continuously recorded with a Datex S/5 monitor unit (S5-Collect, Datex Ohmeda, Duisburg, Germany). Intravascular pressures were referenced to mid-chest level. Cardiac output (CO) was assessed by single indicator transpulmonary thermodilution. For blood-gas analyses, a Rapidlab 248 device (Bayer Healthcare, Leverkusen, Germany) was used (arterial and mixed venous blood gas

analyses). Body temperature was measured via rectal probe and maintained between 37.5 and 38.5 °C by body surface warming. After completing the protocol, the animals were euthanized in deep anaesthesia by injection of 40 mmol potassium and 20 mg/kg propofol.

Details of Study Protocol

After BLH measurements, model lung injury was induced – according to, yet slightly modified from, a previous report on repetitive lung lavages [1] – to study c-R/D. A lavage bag filled with 30 ml/kg of isotonic solution (Sterofundin, B.Braun Melsungen AG, Germany) was connected to the endotracheal tube and mounted 1.5 meter above the subject for fluid administration. Thereafter, placing the bag on the floor passively drained the administered fluid along with the dissolved surfactant. After 30 minutes of stabilization, the Horowitz-index was assessed at an inspired oxygen fraction (F_{IO_2}) of 1.0 by standard arterial blood gas analysis (RapidLab 415, Bayer Healthcare, Germany) at the PEEP settings of maximal derecruitment (PEEP of 0 cm H_2O; ZEEP) and maximal recruitment (PEEP of 15 cm H_2O), respectively. If at ZEEP the Pa_{O_2}/F_{IO_2} ratio was still > 300 and at a PEEP of 15 cm H_2O the Pa_{O_2}/F_{IO_2} ratio was > 450, another lavage was performed. This procedure was repeated until the Pa_{O_2}/F_{IO_2} had decreased to < 300 at ZEEP, or if maximal recruitment had become compromised (Pa_{O_2}/F_{IO_2} < 450 at PEEP 15 cm H_2O). Subsequently, the animals were stabilized for another 30 minutes and analogous to BLH; measurements during model lung injury were recorded at randomized PEEP levels (LAV 0; LAV 5; LAV 10; LAV 15). The animals remained in dorsal recumbency for the entire experiment.

The duration of the protocol was as follows: approximately 180 minutes for induction of anaesthesia and instrumentation; 80 minutes for BLH measurements (four PEEP levels at approximately 20 minutes each); subsequent induction of lung injury, ranging from 70 to 180 min, and 80 min for measurements after lavage.

Time-synchronization of all recording systems

To time-synchronize the different measuring devices, a local area network between the different recording devices was set up, and using a time-server unit, we adjusted all system times to synchronize with the time stamp of the CT-scanner. Moreover, we were able to verify this approach, because the time stamp set by the time-server (when CT acquisition started) correlated perfectly with the change in frequency characteristics above 2000 Hz induced by CT acquisition noise (for more details see paragraph "*Influence of CT acquisition noise on lung sound recordings*"). Although all attempts were made to synchronize the different recording methods, we cannot preclude a minimal temporal offset caused by CT image processing time. However, this seems of minor relevance, as a normal CT scanner makes images available at the same magnitude as the acquisition time.

Preliminary experimental tests

Before carrying out the study, the influence of the surrounding noise on the attached piezoelectric contact sensors and the influence of these upon the radiologic imaging quality were addressed. Using a noise-absorbing mat wrapped around the subjects, pre-testing showed that the recorded raw data sound waveforms were not noticeably affected.

Influence of acoustic sensors on CT imaging

To identify the impact of the acoustic sensors (Meditron, ASA, Norway) on computed tomography (CT) imaging quality, multiple test scans were performed using a plastinated lung (i.e., a plastic casting of a real lung). The most affected lung cross-section with the sensors attached was identified and compared to the identical lung cross-section after removal of the sensors. We found a bias of up to 40 Hounsfield units (HU) for mean lung densities (MLDs) in CT imaging. Fig. S1 shows exemplarily the impact of the acoustic sensors attached.

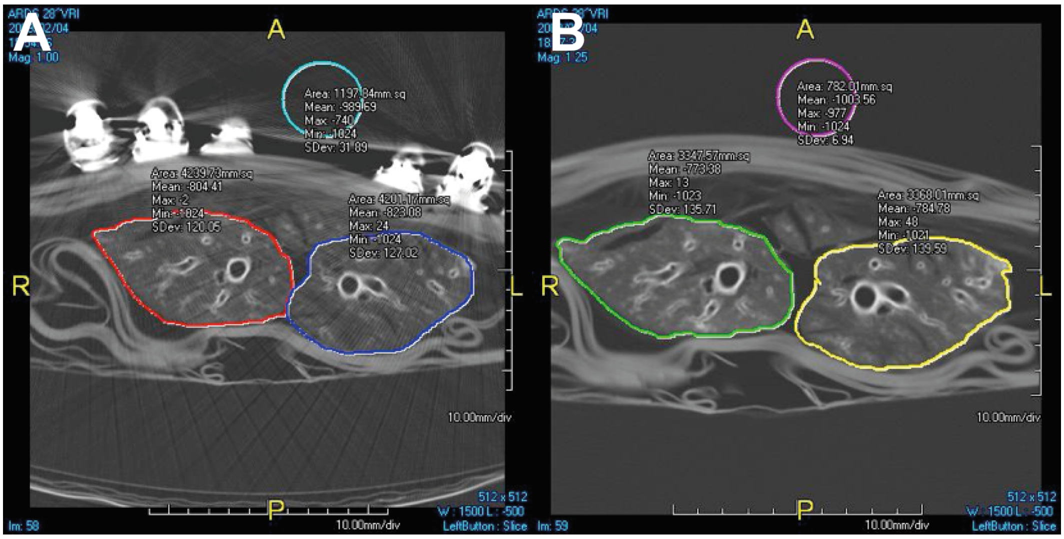

Fig. S1 Impact of the metallic acoustic sensors on mean lung density. Pretesting using a plastinated lung found a bias of up to 40 Hounsfield units (HU) for mean lung densities (MLDs) in CT imaging. Panel A shows the most affected lung cross-section with the sensors attached, Panel B shows the identical lung cross-section after removal of the sensors.

Moreover, we performed a post-hoc analysis of the 4DCT data sets from two piglets to assess the impact the metallic sensors might have had in regard to the different lung regions analyzed, and in regard to lung movement during respiration. Here we found that the impact of the metallic sensors did not significantly differ between the different lung regions of interest, when analyzing the entire lung stack with a longitudinal coverage of 8 cm (non-dependent, central, dependent lung). Further, when looking at the dependent lung (where cyclical recruitment and derecruitment took place), no significant differences were found in the noise caused by the acoustic sensors in the end-expiration lung stack versus the noise in the end-inspiration lung stack. Of course, we observed movement (and changes in noise) caused by mechanical ventilation, but there was nearly no movement in the spinal region of the chest, due to anatomical reasons. This fact, and the fact that we used a saline lavage model injury that produced atelectasis mainly in the dependent part of the lung, might best explain our findings. However, we cannot exclude the possibility that the noise present in the CT signal (which may have been due to these sensors) and the interference caused by movement during mechanical ventilation might have biased the weights of the results and statistics when analysing the complete 4DCT data sets.

Influence of CT acquisition noise on lung sound recordings

We also tested for alterations in the spectra of the recorded lung sounds which might have been induced by CT acquisition noise. Our results showed that CT acquisition noise caused a distinct sound signature above 2000 Hz in frequency, although a noise-absorbing mat was used. However, we want to point out that this exerted no significant influence upon our post-processed sound parameters (FFT area and dCE), as exemplarily highlighted in Fig. S2.

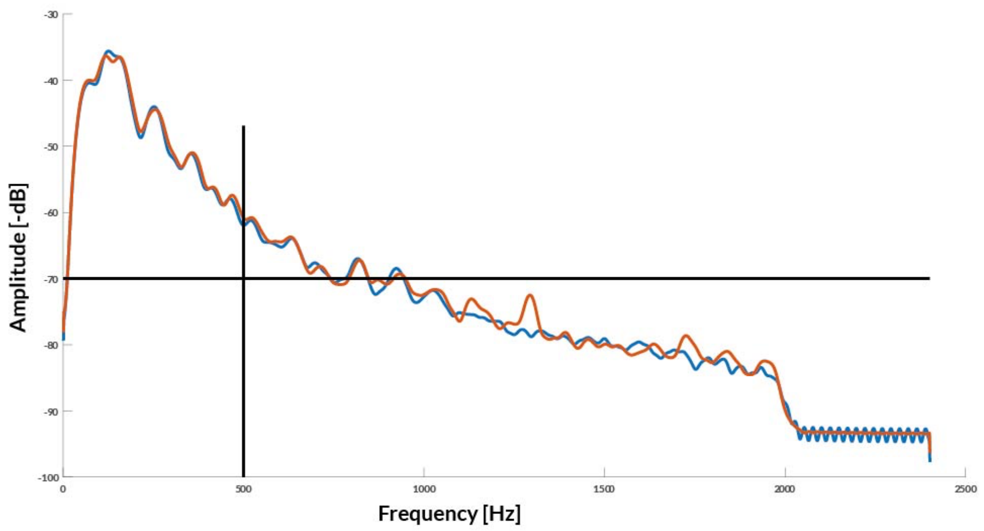

Fig. S2 Impact of CT acquisition noise on lung sound spectra. Example of the resulting sound spectra (frequency amplitude plot) without and during CT acquisition. The blue curve shows the spectrum of the measured lung sounds without the CT running; the orange curve shows the spectrum with the CT running. As one can see, there is an almost perfect agreement between the two spectra – although these were two consecutive breaths.

Details of 4DCT lung segmentation

Lung segmentation and 4DCT analysis was carried out using a specialized software system for analyzing lung CT images, called YACTA, which was developed by one of our authors (OW). The software contains a specific module for semi-automatic and/or full automatic segmentation and quantification of porcine lung 4DCT data.

In our study, one single 4DCT covers 40 CT volumes, each containing 40 CT slices – resulting in 1600 images per 4DCT data set. Due to this large amount of data, a software-based support system was mandatory for the evaluation of data.

In the following, the 3D segmentation and quantification scheme for a 4DCT data set - as implemented in YACTA - is described in a nutshell. The segmentation of the atelectatic areas of the lung is a very demanding task, since the density values of these areas are only very slightly different from the surrounding structures - sometimes there is no difference in the density values at all. Our study possesses two major advantages: Firstly, we have the CT volumes over the entire breathing cycle (40 volumes). Secondly, the volumes are at the same spatial position. There is of course movement caused by mechanical ventilation, but there is nearly no movement in the spinal region of the chest; the movement increases as one progresses to the ventral regions of the chest.

To take advantage of the first point, we searched for the CT volume where the contrast between lung tissue and surrounding tissue was maximized. To accomplish this, the complete 4DCT data set was loaded into the YACTA software, and the CT volume with the minimal mean density (Minimum Density Stack, MDS) was identified (see Fig. S3A). Because of this selection, the MDS should be the easiest volume to be segmented out of all volumes in the time series (e.g., please compare Fig. S3A and S3B). In this volume, the following operations were performed for lung segmentation: First, a Gauss filter was applied; then, high-density structures (≥200 HU; most likely representing bone) and extracorporeal areas (≤ -900 HU) were identified and discarded (Fig. S3B). Thereafter, the N6-connected low-density area (≤ -400 HU) covering the highest volume inside the body was identified and labeled as „Lung". This lung segmentation was refined by morphologic hole-filling and closing algorithms. Final lung segmentation results are shown in Fig. S3C. The fully automatic segmentation results of the lung were then manually refined by our radiologists (YY, TA) using a graphic interface and a painting pencil, in case the region did not completely match the anatomical lung region. However, manual changes were only necessary in a few cases.

Thereafter, the following steps utilized the second advantage of our study. All volumes of one 4DCT series were at the same spatial position, and additionally, the atelectatic changes in the pulmonary tissue occurred mainly in the dorsal lung areas (see Fig. S3E) – in which almost no movement takes place throughout respiration. The lung mask generated for the MDS was transferred and adjusted to all other CT volumes belonging to the 4DCT data set. To adjust the lung borders to correlate most realistically to a specific CT volume, voxels that were within 10 mm distance to the outer border of the previously labeled „Lung" were marked and classified as uncertain voxels (see Fig. S3D). Fig. S3E shows the lung contour (red line) determined in the MDS that was transferred to another CT volume of the 4DCT data set. One can see in Fig. S3E rather nicely that there is almost no movement in the dorsal area, where atelectatic changes are clearly visible. On the other hand, the transferred lung contour does not match very well in the ventral regions of the lung - but there we can still see a higher

contrast between lung tissue and surrounding tissue – and thus, automatic adjustment of the lung contour could be easily performed in these more ventral regions.

The actual adjustment of the transferred lung contour was achieved by the following operations: The bounding box for the entire label "Lung" was calculated and the uncertain "Lung" voxels were classified as follows: In the lower 20% of the box, they were simply marked as „Lung“ (as in these dorsal regions, nearly no movement in body contours occurred over time). In the middle region of the bounding box, uncertain voxels were marked as „Lung“ if HU values were ≤ -200; in the upper region of the bounding box, they were marked as „Lung“ if HU values were ≤ -100). Fig. S3F shows the final adjusted lung contour.

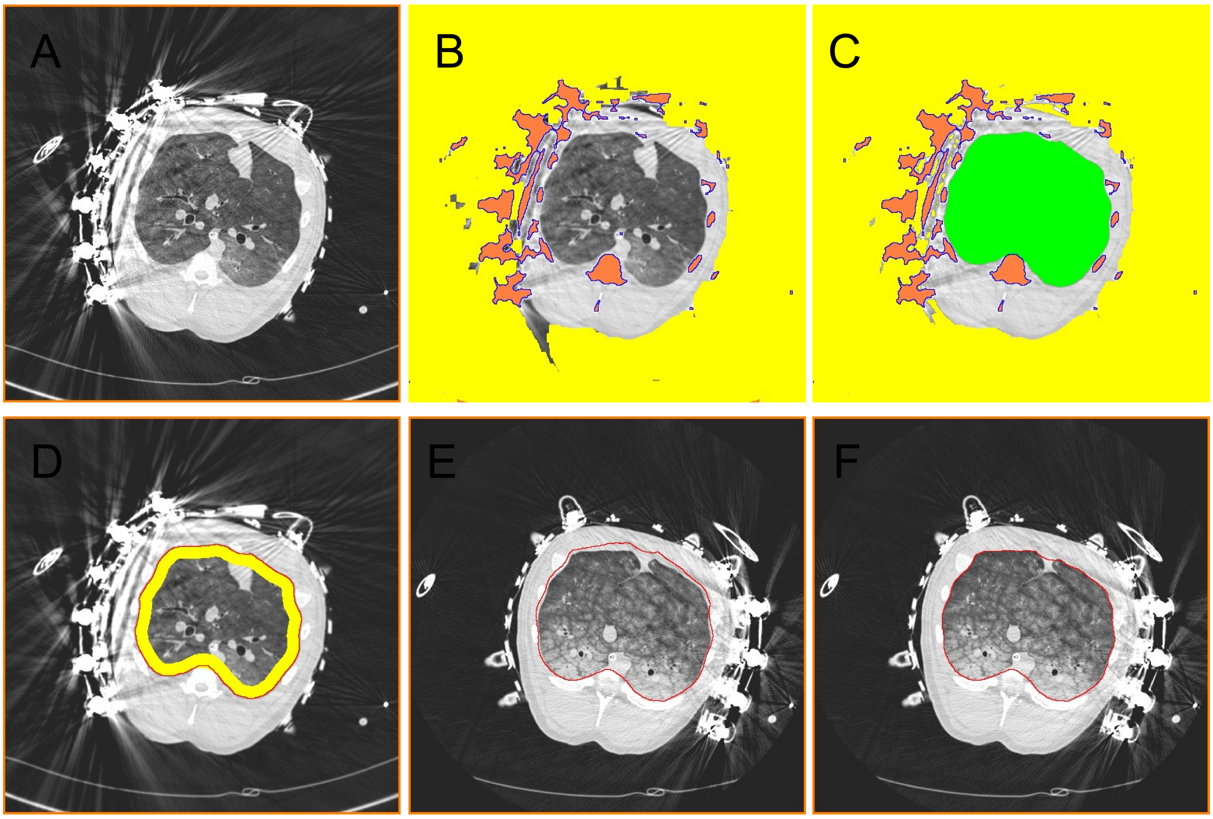

Fig. S3 Example of lung segmentation with the YACTA software. Panel A shows the detected minimal mean density (Minimum Density Stack, MDS) CT volume. Panel B shows the result after having discarded high-density structures and extracorporeal areas. Panel C shows the final lung segmentation result. Panel D shows how the software adjusted the lung mask to all other CT volumes of the 4DCT data set. Here, voxels that were within a 10 mm distance to the outer border of the previously labelled „Lung“ were marked and classified as uncertain voxels. Panel E shows the lung contour determined in the MDS transferred to another CT volume of the 4D data set, and Panel F shows the final adjusted lung contour using a bounding box analysis.

Further, we performed a regional sub-analysis by dividing the marked „Lung“ into equal thirds: The upper third covered the non-dependent lungs areas, the middle third covered the central lung areas, and the lower third the dependent lung areas.

Finally, for each lung region of interest, mean HU and the time course in HU were generated for the mean lung stack and the respective volumes (cm^3) of either atelectatic (-300 to 0 HU), poorly ventilated (-600 to -301 HU), normally ventilated (-900 to -601 HU) or hyperinflated (-1024 to -901 HU) lung areas. Fig. S4 shows an example of 4DCT post-processing.

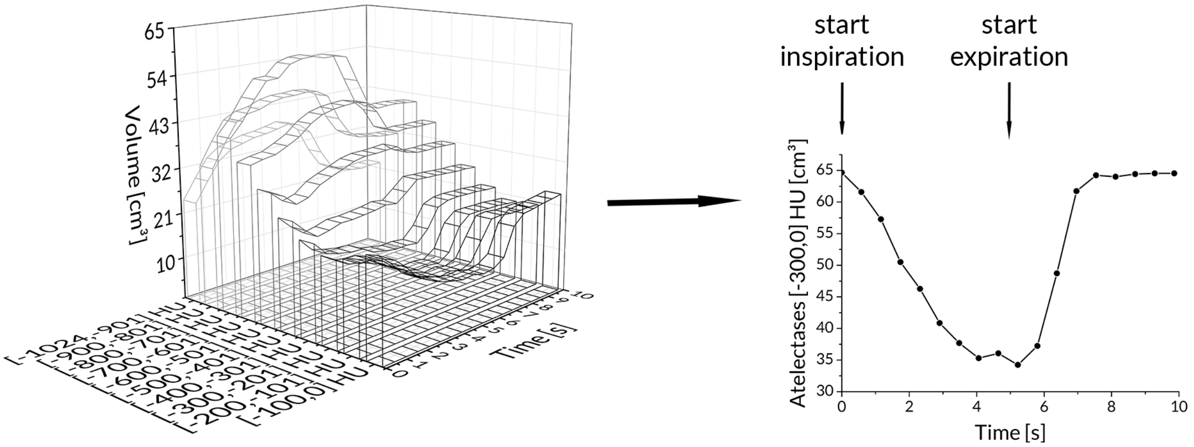

Fig. S4 Post-processing of 4DCT. Exemplary results of 4DCT showing the lung volumes for the defined thresholds in Hounsfield Units over one inspiratory cycle and the post processed changes in atelectatic volume (-300 to 0 HU).

Details of lung sound post-processing

All lung sound analyses (dCE and FFT area) were primarily carried out for each acoustic sensor and in time clips of 0.58 seconds to match the demand acquisition time of 4DCT scanning, resulting in paired measurements over the entire breathing cycle. For final statistics, results were subsumed for the inspiratory phase of the entire (global) lung, and regionally for dependent, central and non-dependent lung areas, by summarizing the parameter values of the respective acoustics sensors overlaying the defined lung regions. All computing was performed using the programming environment MATLAB and Simulink Toolbox Release 2014b (The MathWorks, Inc., Natick, Massachusetts, USA).

An example of the post-processed "FFT area" plot, showing the power spectral density over frequency, is presented in Fig. S5.

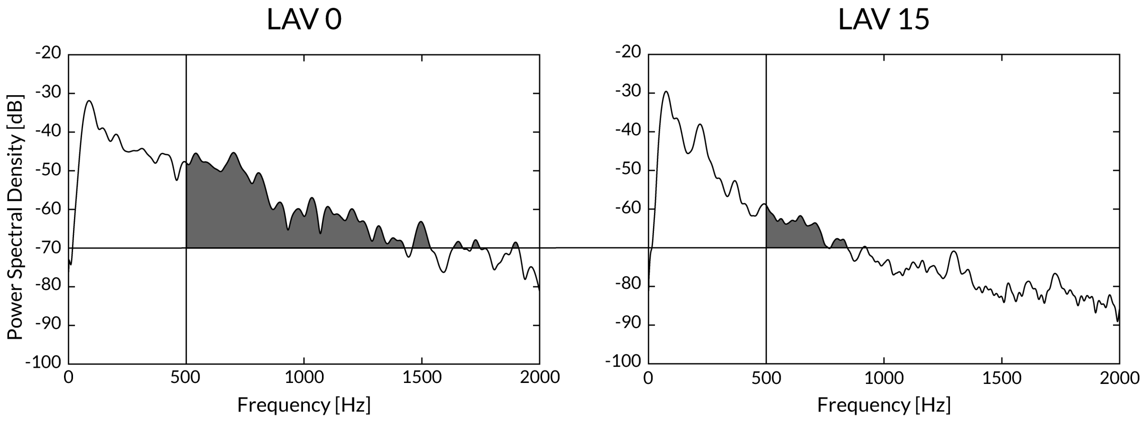

Fig. S5 Example of post-processed power spectral density over all frequencies in lavaged lungs. For the assessment of FFT area, the area under the curve (AUC) above -70 dB in amplitude and above 500 Hz in frequency was related to the AUC above -70 dB and expressed as percentage. At left: ZEEP (LAV 0); at right: at PEEP 15 cm H_2O (LAV 15).

Details of spectral coherence method

Our intent in performing this evaluation was to identify the sensors overlying the lung regions where cR/D takes place. The spectral coherence method was chosen based on the global consideration that fine crackle sounds arise through the sudden recruitment of airways and alveoli, resulting in avalanches of adventitious sounds.

To detect the accompanying changes of spectral characteristics in frequencies over time, assessment of the coherence of neighbouring sensors is a commonly used method to identify how well one input signal corresponds to another input signal.

To gain a parameter representing global similarity between the two signals, we summarized the coherence values over the frequency band. Of two neighbouring sensors Lx.My and Lx.M(y+1) the magnitude squared of the mean cross-spectral density Lx.C_{xy}y(f) was assessed. For each frequency, this estimated cross-spectral density with values between 0 and 1 indicates how well one input signal corresponds to another input signal at each frequency – whereby 1 indicates a strictly linear transfer function between both input signals, and 0 indicates linearly unrelated or uncorrelated signals.

For a global interpretation of the dependency over the entire frequency band, the sum $\sum(Lx.C_{xy}y(f))$ of the $Lx.C_{cxy}y(f)$ function was processed. Thus, for each sensor pair, the result of cross-coherence over the entire frequency band could be reduced to one decimal number/value – with a high value indicating high similarity. Technically, the range for this parameter is 0 to 200 AU (due to the window length of 200 samples), where 0 AU indicates no correlation and 200 AU indicates perfect correlation of the respective acoustic sensors.[2] Finally, according to the subregions (ROIs) investigated, the mean of the respective sensor pairs overlying these regions was calculated for the non-dependent (L1 and L2), central (L3 and L4) and dependent (L5 and L6) areas, and saved in time clips of 0.58 s. From this resulting coherence over time plot (Fig. S6), the mean (spectral choherence; SC) and the standard deviation – variation over time – (dynamic spectral coherence; dSC) was finally post-processed over the inspiratory phase of the breathing cycle.

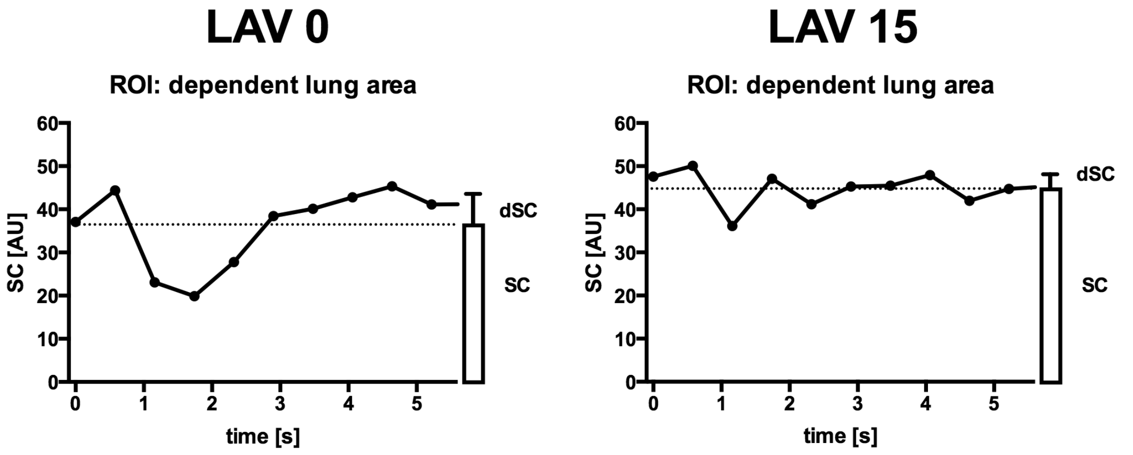

Fig. S6 Example showing the time course of the spectral coherence method over the inspiratory phase of the breathing cycle in the dependent lung after lavage injury. Left at ZEEP (LAV 0), right at a PEEP of 15 cm H_2O (LAV 15). The integrated bar-plots represent the computed parameters of (1) “spectral coherence” (SC), by averaging over the inspiratory phase, and (2) the “dynamic spectral coherence” (dSC), by assessing the standard deviation over inspiration. Increasing PEEP led to a significant reduction in dSC, reflecting less time-dependent variation of spectral coherence between neighbouring acoustic sensors. Concomitant, absolute SC values increased, indicating a restoration of lung homogeneity.

Supplemental Results

Details of 4DCT results

The results for the different PEEP levels of 0, 5, 10 and 15 cm H_2O in the lavaged lungs (LAV 0, LAV 5, LAV 10, and LAV 15) are shown in Fig. S7. While the graphs on the left represent the overall lung volumes in regard to the lung region, the graphs on the right show the variation in volumes (Δ volume) within the respiratory cycle.

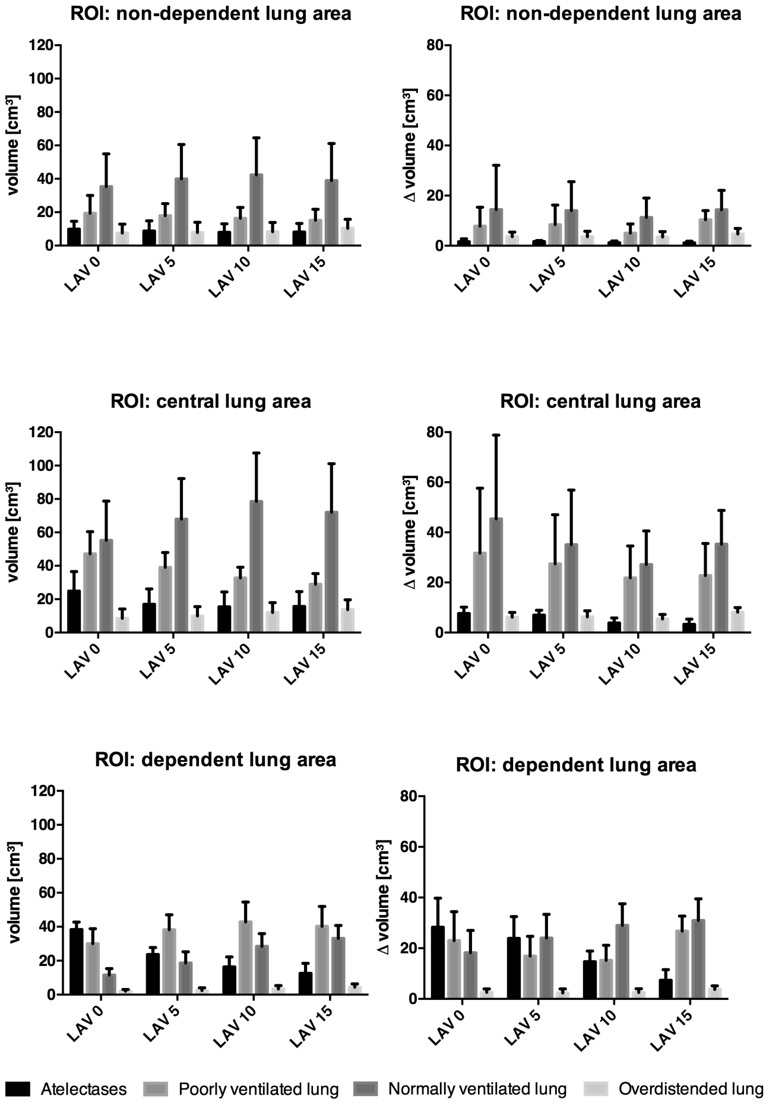

Fig. S7 Showing the amount of atelectatic lung volumes as measured by 4DCT. Results are displayed for mean atelectatic volumes (atelectasis) and for the within-breath changes in atelectatic volumes (Δ atelectasis) during on-going mechanical ventilation after lavage injury. Results are presented as bar charts (means ± standard deviation) for all animals (N=6) and for non-dependent, central and dependent lung regions of interest (ROI), itemized for the respective PEEP settings of 0, 5, 10, and 15 cm H_2O (LAV 0-15).

mean volume [cm³] (mean ± SD)

ROI: non-dependent lung

	Atelectasis	poorly ventilated	normally ventilated	Overdistended
**LAV 0**	9.82 ± 4.73	19.39 ± 10.64	35.33 ± 19.58	7.51 ± 5.3
**LAV 5**	8.74 ± 6.08	17.93 ± 7.26	39.92 ± 20.61	7.88 ± 6.09
**LAV 10**	7.96 ± 5.11	16.33 ± 6.54	42.34 ± 22.26	8.39 ± 5.45
**LAV 15**	8.11 ± 5.4	15.17 ± 6.48	38.94 ± 17.35	10.33 ± 6.89

ROI: central lung

	Atelectasis	poorly ventilated	normally ventilated	Overdistended
**LAV 0**	24.81 ± 11.7	47.12 ± 13.37	55.23 ± 23.55	8.58 ± 5.56
**LAV 5**	16.92 ± 9.27	39.08 ± 8.89	67.92 ± 24.35	9.9 ± 5.69
**LAV 10**	15.43 ± 8.88	32.76 ± 6.39	78.51 ± 29.08	12.01 ± 5.93
**LAV 15**	15.63 ± 8.95	28.98 ± 4.46	72.04 ± 19.92	13.74 ± 6.06

ROI: dependent lung

	Atelectasis	poorly ventilated	normally ventilated	Overdistended
**LAV 0**	38.32 ± 4.44	30.01 ± 8.8	11.57 ± 3.81	2.03 ± 1.07
**LAV 5**	23.64 ± 4.08	38.24 ± 8.77	18.69 ± 6.6	2.46 ± 1.56
**LAV 10**	16.4 ± 5.83	42.85 ± 11.69	28.44 ± 7.55	3.39 ± 1.93
**LAV 15**	12.59 ± 3.72	40.27 ± 9.3	33.2 ± 9.78	4.44 ± 2.21

Table S1 Mean lung volumes as measured by 4DCT. Results are displayed for the average volumes of hyperinflated, normally aerated, poorly aerated and atelectatic lung after lavage injury. Measures are given for non-dependent, central and dependent lung regions of interest (ROI), itemized for the respective PEEP settings of 0, 5, 10, and 15 cm H_2O (LAV 0-15), as mean and standard deviation for all animals.

Table S1 and Table S2 shows detailed results of the lung volumes classified as hyperinflated, normally aerated, poorly aerated and atelectatic lung areas, as mean and SD for all animals after lavage injury. Table S1 represents the average lung volumes; Table S2 represents the within-breath changes during ongoing mechanical ventilation. Measures are given for non-dependent, central and dependent lung regions of interest (ROI), itemized for the respective PEEP settings of 0, 5, 10, and 15 cm H_2O (LAV 0-15).

Δ volume [cm³] (mean ± SD)				
**ROI: non-dependent lung**				
	**Atelectasis**	**poorly ventilated**	**normally ventilated**	**Overdistended**
**LAV 0**	1.61 ± 1.19	7.85 ± 7.54	14.46 ± 17.63	3.62 ± 1.81
**LAV 5**	1.69 ± 0.42	8.42 ± 7.82	14.05 ± 11.49	3.61 ± 2.13
**LAV 10**	1.26 ± 0.64	5.01 ± 3.7	11.3 ± 7.73	3.5 ± 2.14
**LAV 15**	1.17 ± 0.38	10.35 ± 11.44	14.36 ± 13.76	4.72 ± 3.41
**ROI: central lung**				
	**Atelectasis**	**poorly ventilated**	**normally ventilated**	**Overdistended**
**LAV 0**	7.63 ± 2.56	31.68 ± 25.94	45.37 ± 33.46	6.16 ± 1.87
**LAV 5**	6.98 ± 1.93	27.45 ± 19.61	35.09 ± 21.8	6.3 ± 2.37
**LAV 10**	3.81 ± 2.05	21.81 ± 12.76	27.16 ± 13.41	5.44 ± 1.79
**LAV 15**	3.31 ± 2.17	22.79 ± 16.9	35.32 ± 31.16	8.14 ± 5.55
**ROI: dependent lung**				
	**Atelectasis**	**poorly ventilated**	**normally ventilated**	**Overdistended**
**LAV 0**	28.27 ± 11.47	23.04 ± 11.41	18.23 ± 8.79	2.65 ± 1.33
**LAV 5**	23.93 ± 8.59	16.91 ± 7.76	24.06 ± 9.32	2.34 ± 1.61
**LAV 10**	14.7 ± 4.21	15.22 ± 5.92	29.03 ± 8.55	2.6 ± 1.42
**LAV 15**	7.34 ± 2.93	26.78 ± 16.51	30.97 ± 16.06	3.77 ± 3.35

Table S2 Within-breath changes in lung volumes as measured by 4DCT. Results are displayed for the within-breath changes (Δ) in hyperinflated, normally aerated, poorly aerated and atelectatic volumes during on-going mechanical ventilation after lavage injury. Measures are given for non-dependent, central and dependent lung regions of interest (ROI), itemized for the respective PEEP settings of 0, 5, 10, and 15 cm H_2O (LAV 0-15).

Details of 4DCT dCE and FFT area results

The variations over the inspiratory cycle of dCE, the FFT area, and changes in atelectatic volume by 4DCT appear in Fig. S8 for the different time-points in the entire lung stack of lavaged animals. As seen in S8 Figure, dCE and FFT area signals predominately arose in the first one to two seconds after the initiation of inspiration, a time period when 4DCT indicated the greatest changes in atelectatic lung volume. Data are presented for the entire lung, and in regard to the respective PEEP levels of 0, 5, 10 and 15 cm H_2O (LAV 0, LAV 5, LAV 10, and LAV 15) of the lavaged lungs.

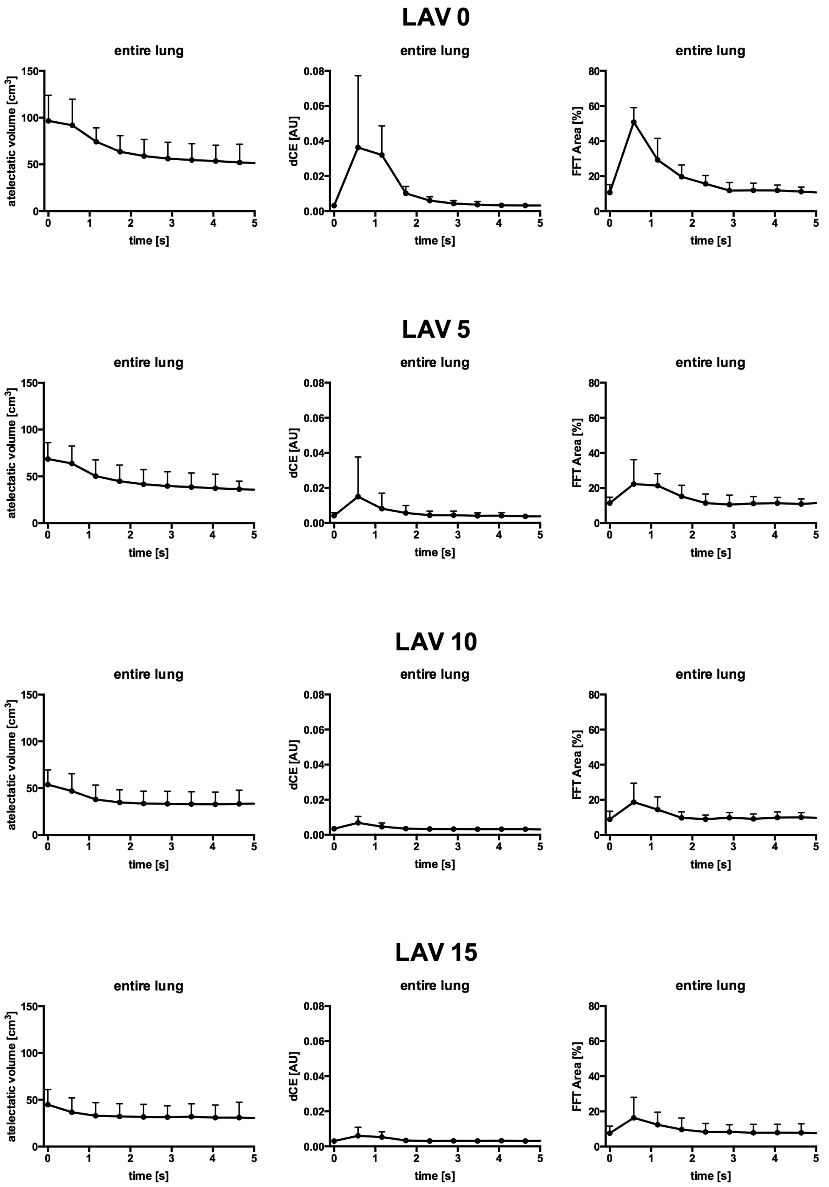

Fig. S8 Showing the time-synchronized measures of Δ atelectasis by 4DCT, dCE and FFT area. Data are presented descriptively as mean ± standard deviation for all subjects (N=6) at ZEEP (LAV 0), a PEEP of 5, 10 and 15 cm H_2O (LAV 5, 10, 15) in model lung injury for the entire lung stack.

Details of "dynamic spectral coherence" results

Estimates with standard error (SE) of the pair-wise comparisons of the fitted ANOVA model are presented in Table S3.

dSC: ANOVA

Effect	df1	df2	F	*P* value
**PEEP**	1	25	10.7	0.0031
**lung region (ROI)**	2	25	11.95	0.0002
**PEEP*ROI**	2	25	1.41	0.2633

dSC: Model-based Estimates of the differences in mean

Effect	ROI	PEEP	ROI	PEEP	Estimates	SE	*P* value
**PEEP**		0		15	1.7537	0.5362	0.0031
**ROI**	dependent		central		1.9403	0.6567	0.0067
**ROI**	dependent		non-dependent		3.1851	0.6567	<.0001
**ROI**	central		non-dependent		1.2448	0.6567	0.0696

Table S3 Results of the analysis of variance (ANOVA) based on the linear mixed model (LMM) of the parameter dSC. dSC: dynamic spectral coherence; PEEP: positive end-expiratory pressure; ROI: region of interest.

Detailed results of "spectral coherence method"

Fig. S9 displays the spectral coherence (SC) and dynamic spectral coherence (dSC) data for the different PEEP levels in regard to lung region after surfactant depletion injury (LAV).

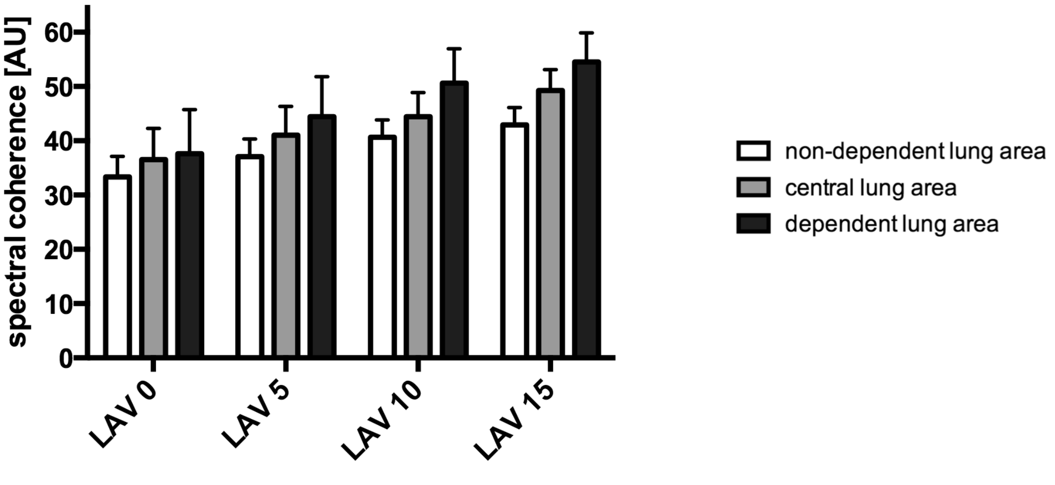

Fig. S9 Spectral coherence and variation over the respiratory cycle. For the different time points at ZEEP (LAV 0), a PEEP of 5, 10 and 15 cm H_2O (LAV 5, 10, 15), the bar-plots represent the computed parameters of (1) "spectral coherence" (SC), by averaging over the inspiratory phase and (2) "dynamic spectral coherence" (dSC), by assessing their variation (standard deviation) over the inspiratory phase of the breathing cycle - itemized for the non-dependent, central and dependent lung regions.

References

1. Boehme S, Bentley AH, Hartmann EK, Chang S, Erdoes G, Prinzing A, et al. Influence of Inspiration to Expiration Ratio on Cyclic Recruitment and Derecruitment of Atelectasis in a Saline Lavage Model of Acute Respiratory Distress Syndrome. Crit Care Med. 2015;43:e65-74.
2. Kay SM. Modern spectral estimation: theory and application. Englewood Cliffs, NJ: Prentice Hall; 1988.
